# Type 2 diabetes does not inevitably shorten life expectancy^☆^

**DOI:** 10.1016/j.mmr.2026.100029

**Published:** 2026-04-27

**Authors:** Huai-Dong Du

**Affiliations:** Clinical Trial Service Unit and Epidemiological Studies Unit, Nuffield Department of Population Health, University of Oxford, Oxford OX3 7LF, UK

**Keywords:** Diabetes care, Mortality, Life expectancy, Lifestyle factors

Type 2 diabetes (T2D) has long been regarded as a chronic condition inevitably associated with premature mortality and reduced life expectancy [1]. Although pharmacological advances have substantially improved glycaemic control and complication prevention, life expectancy loss is still widely perceived as unavoidable. In this context, the recent study by Qiu *et al*. [2] (hereafter “the Study”), published in the *Military Medical Research*, provides timely and compelling evidence that fundamentally challenges this long-standing perception. Leveraging three large nationwide prospective cohort studies from China, the United States, and the United Kingdom, the authors demonstrated that people with T2D who achieve control of at least 5 out of 6 major lifestyle (smoking, physical inactivity, and unhealthy diet) and metabolic [elevated glycated haemoglobin (HbA1c), dyslipidaemia, and high blood pressure] risk factors can attain a life expectancy comparable to that of adults without diabetes. Notably, observed longevity benefits were independent of genetic predisposition to a shorter lifespan. The Study offers several important innovations. Firstly, it examined 6 modifiable risk factors simultaneously, allowing direct comparison of their relative contributions. Secondly, it estimated life expectancy rather than relying solely on hazard ratios, translating epidemiological associations into clinically meaningful years of life gained or lost. Finally, the consistency of findings across three distinct national populations enhances generalisability.

## Lifestyle behaviours as the cornerstone for diabetes management

One of the most striking findings of the Study is the dominant role of lifestyle behaviours in determining the life expectancy of T2D patients. Across all cohorts, adherence to healthy lifestyle behaviours was consistently associated with substantial reductions in mortality risk and meaningful gains in life expectancy (i.e., 3−6 years), regardless of metabolic risk factor control, with dietary quality as the single most influential risk factor (i.e., achieving a healthy diet associated with life expectancy gains of approximately 2.6−4.1 years). This finding is particularly important given the long-standing emphasis on pharmacological glycaemic control over dietary and other lifestyle modification in diabetes management. While diet and physical activity are universally recommended as part of diabetes care, their long-term impact on “hard” outcomes such as mortality and life expectancy has been less clearly quantified. Previous studies evaluating the impact of lifestyle interventions on T2D management have typically focused on intermediate metabolic outcomes (e.g., HbA1c, lipid profiles, and blood pressure) [3], [4], limiting direct comparisons of relative importance between lifestyle behaviours and metabolic control. The Study therefore provides rate and persuasive evidence, suggesting that lifestyle modification (particularly improvements in dietary quality) should be emphasised, even in the era of modern cardioprotective glucose-lowering agents. From a clinical perspective, these findings suggest that risk factor prioritisation in T2D management warrants reconsideration. While glycaemic control remains essential, well-structured, evidence-based lifestyle counselling should be integrated as a central component of routine diabetes care rather than being treated as an adjunct recommendation. Health systems should invest in dietary education, behavioural support, and long-term lifestyle maintenance strategies to optimise diabetes care.

## Future research directions and policy implications

The Study is methodologically robust, with findings being well-interpreted. The use of life expectancy as a health outcome provides an intuitive and patient-centred measure that complements traditional relative risk estimates. Nonetheless, the role of body weight and adiposity needs to be investigated and fully appreciated in the Study. Despite the non-linear (i.e. J-shaped or U-shaped) observational association between adulthood body mass index (BMI) with all-cause or cause-specific mortality (often referred as “obesity paradox”), due largely to the impact of reverse causality and residual confounding [5], the survival benefits associated with maintaining a healthy BMI in the general population and among people living with diabetes have been well established [6]. Moreover, sustained weight loss has been recognised as a dominant driver of diabetes remission in randomised clinical trials [7]. Accordingly, major international diabetes management guidelines, including those from the American Diabetes Association (ADA), Diabetes UK and the International Diabetes Federation (IDF), consistently emphasise the importance of body weight control as a core component of diabetes care. Future analyses building on the Study could explore whether adiposity status across the three cohorts partially explains the observed variation in target achievement rates, and whether the greater survival benefit related to lifestyle risk factor control (i.e., relative to metabolic risk factor control) is mediated by adiposity and body weight control. Incorporating adiposity measures into risk score calculation may clarify whether maintaining a healthy body weight confers additional life expectancy benefit in T2D patients. Such evidence would further strengthen the rationale for, and promote the adherence to, population-level obesity prevention initiatives, such as the China Healthy Weight Management Action Plan launched in 2025.

Although genetic predisposition to early death did not modify the survival benefits observed in the Study, accumulating evidence suggests that uniform, “one-size-fits-all” approaches to disease prevention and control may overlook substantial inter-individual differences in risk profiles, treatment response, and disease trajectories. Therefore, differentiated strategies by age, sex, adiposity status, socioeconomic context and other individual characteristics (e.g., gut microbiome composition) are needed to maximise the effectiveness of secondary (and primary) T2D prevention. Expanding research to include non-fatal outcomes (e.g., cancer, dementia, infectious diseases and multimorbidity) would further inform the development of well-targeted cost-effective strategies for T2D management.

## Emerging pharmacological advances and future expectations

Recent years have witnessed major advances in obesity and diabetes pharmacotherapy, such as semaglutide and tirzepatide [8], [9]. These agents have demonstrated unprecedented efficacy in both glycaemic control and sustained weight reduction, with additional cardiovascular and renal protective effects in large-scale randomised controlled trials. Viewed alongside the Study’s findings, we could reasonably expect that combining structured lifestyle interventions with highly effective weight-loss and glucose-lowering agents may further reduce the health risk of T2D. However, pharmacological innovation should complement, not replace, healthy lifestyles. Future research should evaluate whether integrating these novel therapies within multifactorial risk-control frameworks translates into additional health gains in life expectancy and reductions in multimorbidity among people with T2D. At the same time, their long-term safety and cost-effectiveness must be carefully monitored.

## Conclusions

The Study provides compelling evidence that T2D does not inevitably shorten life expectancy. Rather, excess mortality in T2D is largely driven by the accumulation of modifiable lifestyle and metabolic risk factors. Achieving comprehensive risk factor control, particularly through healthy lifestyle behaviours (i.e., a healthy diet), can substantially narrow or even eliminate the life expectancy gap between individuals with and without diabetes [1]. These findings have profound implications for clinical practice and public health policy, underscoring the central role of healthy lifestyles and effective risk factor management in people with T2D. They call for a paradigm shift in diabetes care, from a predominantly pharmaco-centric model to one that places lifestyle modification at the core of prevention and long-term management strategies.

## Abbreviations

T2D: Type 2 diabetes

BMI: Body mass index

HbA1c: Glycated haemoglobin

## Ethics approval and consent to participate

Not applicable

## Funding

Not applicable

## Data Availability

Not applicable
